# Differential Properties of Human ALP^+^ Periodontal Ligament Stem Cells vs Their ALP^-^ Counterparts

**DOI:** 10.4172/2157-7633.1000292

**Published:** 2015-07-16

**Authors:** Zongdong Yu, Philippe Gauthier, Quynh T Tran, Ikbale El-Ayachi, Fazal-Ur-Rehman Bhatti, Rayan Bahabri, Mey Al-Habib, George TJ Huang

**Affiliations:** 1 Department of Bioscience Research, University of Tennessee Health Science Center, College of Dentistry, Memphis, USA; 2 Department of Endodontics, Henry M. Goldman School of Dental Medicine, Boston University, Boston, USA; 3 Département d’endodontie, Faculte de medicine dentaire, Université Laval, Quebec, QC, Canada; 4 Department of Preventive Medicine, College of Medicine, Memphis University of Tennessee Health Science Center, USA

**Keywords:** Human periodontal ligament stem cells, PDLSCs, Mesenchymal stem cells, MSCs, Alkaline phosphatase, ALP, STRO-1, CD146, Stemness genes, OCT4, NANOG, SOX2, Subpopulation, Osteogenic induction, BSP, RUNX2, OCN, Adipogenesis, Chondrogenesis, Neurogenesis

## Abstract

Characterizing subpopulations of stem cells is important to understand stem cell properties. Tissue-nonspecific alkaline phosphatase (ALP) is associated with mineral tissue forming cells as well as stem cells. Information regarding ALP subpopulation of human periodontal ligament stem cells (hPDLSCs) is limited. In the present study, we examined ALP^+^ and ALP^−^ hPDLSC subpopulations, their surface markers STRO-1 and CD146, and the expression of stemness genes at various cell passages. We found that ALP^+^ subpopulation had higher levels of STRO-1 (30.6 ± 5.6%) and CD146 (90.4 ± 3.3%) compared to ALP^−^ (STRO-1: 0.5 ± 0.1%; CD146: 75.3 ± 7.2%). ALP^+^ cells expressed significantly higher levels of stemness associated genes, *NANOG, OCT4* and *SOX* than ALP^−^ cells at low cell passages of 2-3 (p<0.05). ALP^+^ and ALP^−^ cells had similar osteogenic, chondrogenic and neurogenic potential while ALP^−^, not ALP^+^ cells, lacked adipogenic potential. Upon continuous culturing and passaging, ALP^+^ continued to express higher stemness genes and STRO-1 and CD146 than ALP^−^ cells at ≥passage 19. Under conditions (over-confluence and vitamin C treatment) when ALP^+^ subpopulation was increased, the stemness gene levels of ALP^+^ was no longer significantly higher than those in ALP^−^ cells. In conclusion, ALP^+^ hPDLSCs possess differential properties from their ALP^−^ counterparts.

## Introduction

Since the isolation and characterization of human periodontal ligament stem cells (hPDLSCs) [1], these cells have been shown to have potential for regenerative purposes [2-4]. Therefore, further understanding of the PDLSC characteristics and properties is important. PDLSCs are a type of mesenchymal stromal/stem cells (MSCs) with multiple differentiation potential [1,5,6]. Tissue-nonspecific alkaline phosphatase (ALP) is commonly known as an early marker gene during osteogenesis and odontogenesis and it plays a role in bone matrix mineralization [7-12]. In vivo studies suggest that PDLSCs may differentiate into cementoblasts and osteoblasts [1,13]. Subpopulation studies of trabecular bone derived cells or bone marrow MSCs have linked the ALP^+^ subpopulation to more differentiated osteogenic cells whereas ALP^−^ cells are more immature cells [14,15]. Of note, ALP is also a marker to indicate the undifferentiated state of embryonic stem cells (ESCs) [16] and induced pluripotent stem cells (iPSCs) [17] besides other pluripotency-associated genes such as *NANOG, OCT4* and *SOX2*. The latter three have also been used to identify the stemness levels of adult stem cells and their expression levels can increase in these adult stem cells after certain treatments [18,19]. Therefore, to further understand the stem cell properties of PDLSCs, it is logical to ask whether ALP^+^/ALP^−^ subpopulations in PDLSCs are associated with cell stemness levels.

STRO-1 and CD146 have been associated with MSC stemness [20,21]. STRO-1^+^ or CD146^+^ subpopulation from human dental pulp stem cells (DPSCs) form more colony-forming units-fibroblastic (CFUs-F) than the heterogeneous pool of DPSCs [22]. The expression of both markers tend to decrease at higher cell passages in cultures [18,23]. CD146-expressing cells in human bone marrow (BM) stroma are capable of establishing hematopoietic microenvironment after transplantation into heterotopic sites [21]. In the present study, we aimed to isolate and characterize ALP^+^ hPDLSCs and examine their correlation with the expression of STRO-1, CD146, and pluripotency-associated genes in comparison to their ALP^−^ counterparts.

## Materials and Methods

### Cell cultures

PDL tissues were removed from extracted third molars (n=27) of healthy patients between 14 and 30 years old in the Oral Surgery Clinics at Boston University (BU) or University of Tennessee Health Science Center (UTHSC). The patient sample collection in this study conformed to exempt protocols approved by the Institutional Review Board (IRB) of BU (#H-28882) and UTHSC (12-01937-XM). The tissues were minced into 1×1×1 mm fragments, digested in collagenase/dispase to obtain single-cell suspensions as described previously [1,24]. The formation of CFU-F was observed and allowed to expand for passaging. Some cultures at passage 0 were stained for the presence of STRO-1. To study growth difference of PDL cells isolated either by enzyme digestion or explant outgrowth, some PDL samples were divided into two halves to isolate PDLSCs by enzyme digestion (PDLSCs-d) or explant outgrowth method (PDLSCs-o). Cells were grown in α-minimum essential medium (α-MEM; Life Technologies/GIBCO BRL, Gaithersburg, MD) supplemented with 10% fetal bovine serum (FBS), 2 mM L-Glutamine, 100 μM L-ascorbic acid-2-phosphate, and antibiotic/antimycotic agents. Human bone marrow (BM)–derived MSCs (BMMSCs) were obtained from Dr. R. S. Tuan (National Institutes of Health, Bethesda, MD) and cultured on the basis of our previous report [25]. Human jaw bone-derived MSCs (JBMSCs) were isolated as described previously [23] and grown in the same medium as for PDLSCs. For passaging, cells were split (1:3 ratio) at ~80% subconfluence.

### Proliferation analysis

For cell proliferation studies of PDLSC subpopulations, cells were sorted at passage 3. Original pool, ALP^+^ and ALP^−^ PDLSCs were then immediately plated at a density of 1,000 cells/well of 12-well plates. Cells were then harvested and counted in a hemocytometer under the microscope at a later time point.

### Immunocytofluorescence analysis

The following mouse anti-human primary antibodies were used for immunocytofluorescence. Mouse anti-human: STRO-1 IgM (Invitrogen), CD73 IgG1 (BioLegend), CD90 IgG1 (BD Pharmingen), CD105 IgG1 (eBioscience, San Diego, CA), CD146 IgG1 (Invitrogen), βIII-tubulin antibodies (Promega corp., Madison, WI), and isotype controls IgG and IgM (both from Invitrogen). Secondary antibodies included goat anti-mouse IgM or IgG1 Alexa Fluor 594 and goat anti-mouse IgG1 Alexa Fluor 488, all from Invitrogen.

Cells grown in chamber glass slides (8 wells) or in culture plates were washed and fixed with 100% ice-cold methanol for 7-10 minutes. After PBS washing, cells were blocked with 5% goat serum in PBS or in blocking buffer (32.5 mM NaCl, 3.3 mM Na2HPO4, 0.76 mM KH2PO4, 1.9 mM NaN3, 0.1% [w/v] bovine serum albumin (BSA), 0.2% (v/v) Triton-X 100, 0.05% (v/v) Tween 20, and 5% goat serum) for 30 min. The primary antibody was then added directly to cells and incubated for 1 hour at room temperature, washed with PBS for 3 times each 5 minutes on a rocker. After PBS wash, secondary antibody (Alexa Fluor 594 or Alexa Fluor 488) in blocking buffer was added and incubated for 1 hour at room temperature in dark. Subsequently, cell nuclei were stained with 4′,6-diamidino-2-phenylindole dihydrochloride (DAPI) for 3 minutes. Images were analyzed under a fluorescence microscope.

### Flow cytometry

Subconfluent cells were harvested for analysis and the antibodies used were the following: FITC-STRO-1 (Biolegend, San Diego, CA), APC-ALP (R&D Systems, CustomerService@RnDSystems.com); PE-CD146; PE-Cy7-CD90; PerCP-Cy^™^5.5-CD73 (the latter three all from BD Pharmingen^™^); V450-CD105 (BD Horizon^™^). Relevant conjugated mouse IgG_1_ (BD Pharmingen^™^) or IgM (Biolegend, San Diego, CA) were used as isotype control.

For direct single staining of cell surface antigens, cell aliquots (2-8 × 10^5^ cells) were washed twice with 1% BSA in phosphate buffered saline ( PBS), re-suspended in a buffer [0.1% FBS in phosphate buffered *saline* (PBS)] or a blocking buffer (10 μg/mL mouse IgG in PBS) for 15 minutes at room temperature, and incubated for 60 min at 4°C in the dark with conjugated PerCP Cy5.5, Cy7, APC. PE, FITC or Alexa Fluor antibodies according to the manufacture’s recommendations. Cells were then washed twice and re-suspended in 1% BSA/PBS for analysis on a flow cytometer (LSRII flowcytometer, BD Biosciences) using the FlowJo X software (BD Biosciences).

For direct quadruple staining, an anti-mouse Ig/negative control compensation polystyrene microsphere set (AbC^™^ Anti-Mouse Bead Kit, Invitrogen, Eugene, OR) was used for the quadruple staining according to the manufacturer’s instruction. Cell aliquots of 2×10^5^ placed in a sample tube were washed twice in a staining buffer (1% BSA), resuspended in a blocking buffer (10 μg/mL mouse IgG in PBS) and incubated for 15 min at room temperature. Four anti-human fluorochrome-conjugated mouse IgM or IgG antibodies -- FITC-STRO-1, APC-ALP, PE-CD146 and PE-Cy7-CD90 of appropriate dilution were all added into the cell sample tube and vortexed. After incubation for 60 minutes at 4°C (in the dark), cells were then washed twice and resuspended in staining buffer for analysis. The negative control compensation polystyrene microsphere set was prepared as follows. Negative beads and the AbC^™^ anti-mouse Ig binding beads were added into empty sample tubes and vortexed. Each conjugated antibody FITC-STRO-1, APC-ALP, PE-CD146 or PE-Cy7-CD90 of the same respective dilution as for staining the cells was then added to a sample tube containing the beads and vortexed. The antibody and bead mixture was incubated at room temperature in the dark for 30 minutes and then washed with staining buffer followed by resuspending in fresh staining buffer for analysis. At the time for each flow cytometry analysis of the quadruple staining, 6 sample tubes were prepared: 1) unstained cells (10^5^), 2) FITC-STRO-1/bead mixture, 3) APC-ALP/bead mixture, 4) PE-CD146/bead mixture, 5) PE-Cy7-CD90/bead mixture and 6) cells stained with all four antibodies. Optimized fluorescence compensation settings for multicolor flow cytometric analyses were performed using a LSR II flow cytometer (BD Biosciences) and the CellQuest ProTM software (BD Biosciences).

### Cell sorting

ALP^+^/ALP^−^ cells were separated by magnetic beads cell separation (MACS Separator kits, MACS Miltenyi Biotec Inc. Auburn, CA) according to manufacturer’s instruction. Cells (10^7^) were harvested and washed in cell sorting buffer (0.5% BSA and 2mM EDTA in PBS, pH 7.2), centrifuged and resuspended in the blocking buffer, same as that used in FACS sorting described below, for 15 min at room temperature. APC-ALP antibody was then added to the cells and incubated in the dark for 60 min at 4°C. Cells were then washed twice in sorting buffer and resuspended in 80 μL sorting buffer. Twenty μL of Anti-APC Microbeads were then added to the cells and mixed well followed by incubation for 15 min at 4°C in the dark. Subsequently, cells were washed in buffer and resuspended in 500 μL sorting buffer and loaded onto a prepared column placed on a separator. The collected cells that flowed through the column were ALP^−^ cells. The column was then removed from the separator and placed on a collection tube. The ALP^+^ cells were collected from the column after adding the buffer and firmly pushing the plunger into the column.

We also applied fluorescence activated cell sorting (FACS) to sort ALP^+^/ALP^−^ cells, 10^7^ cells were washed twice with sorting buffer (5% FBS in culture medium), resuspended in a blocking buffer (10 μg/mL mouse IgG in PBS) and incubated for 15min at room temperature. Mouse anti-human ALP (APC-ALP) antibody of appropriate dilution was added into the cells and vortexed. After incubation for 60 minutes at 4°C (in dark), cells were washed twice in a sorting buffer (5% FBS in cell culture medium) and resuspended in the same buffer for FACS (BD Biosciences FACSAria Cell Sorter). Sorted cells were collected in a collecting buffer (20% FBS in culture medium), further cultured, frozen down or passaged until experimentation. We found that using FACS or MACS yielded similar results. In the present study, FACS was used for data in Figure 3C, all other data required sorting were from the MACS method.

### Lineage differentiation

#### Osteogenic differentiation

Cells were seeded into 12-, 24-or 48-well plates, grown to ~70-80% confluence and incubated in differentiation medium containing 10 nM dexamethasone, 10 mM β-glycerophosphate, 50 μg/ml ascorbate phosphate, 10 nM 1, 25 dihydroxyvitamin D_3_ and 10% FBS for various time points and then processed for analyses. The medium was changed every 3 days. Cultures were harvested for qPCR, or fixed in 60% isopropanol, and mineralization of extracellular matrix stained with 1% Alizarin red S (ARS) [24]. For quantitative analysis, the ARS stained cultures were dissolved in cetylpyridinium chloride (CPC) buffer (10% CPC (w/v) in 10mM Sodium Phosphate Buffer, Sigma) for 1 hour. Three aliquots in 200 μL of ARS/CPC extract from each well were then transferred to a 96-well reading plate and quantified by absorbance measurement at 550 nm by a spectrophotometer (Bio-Rad).

#### Adipogenic differentiation

Cells were seeded in 12- or 48-well plates and grown to subconfluence as for osteo-induction, and incubated in adipogenic medium containing 1 μM dexamethasone, 1 μg/ml insulin, 0.5 mM 3-isobutyl-1-methylxantine (IBMX) and 10% FBS for 6-8 weeks. The medium was changed every 3 days. Cultures were harvested for qPCR or fixed in 10% formalin for 60 min, washed with dH_2_O and 60% isopropanol, and lipid droplets were stained with 0.18% (w/v) Oil Red O reagent for 5 min and washed with water.

#### Chondrogenic differentiation

Cells were seeded into 24-well plates or into 200 μl microfuge tubes and centrifuged down to form cell pellets. Cell cultures in plates or in tubes were treated for 3 weeks with chondrogenic medium, consisting of high-glucose DMEM supplemented with 100 nmol/L dexamethasone, 50 μg/ml ascorbic acid-2-phosphate, 100 μg/ml sodium pyruvate, 40 μg/ml l-proline, 10 ng/ml recombinant human transforming growth factor-β3 (TGF-β3; R&D Systems, Minneapolis, MN), and 50 mg/ml ITS-premix stock (BD Biosciences). The medium was changed every 3 days. Chondrogenic cell cultures or pellets were fixed in 4% buffered paraformaldehyde for 60 min, washed and stained with Alcian blue (pH 3.0) to detect sulfated proteoglycans.

#### Neurogenic differentiation

Cells at subconfluence in chamber slides or in 12- or 24-well plates were stimulated by one of the two induction protocols. i) Neurogenic induction medium (NIM-1) consisted of Neurobasal A (Gibco-Invitrogen) with B27 supplement (GIBCO-BRL), 20 ng/mL epidermal growth factor (EGF) (BD Biosciences), and 40 ng/mL fibroblast growth factor (FGF) (BD Biosciences) was added to the cells and cultures incubated for 4 weeks with the medium refreshed every 3 days. Cultures were then analyzed by immunocytofluorescence for the expression of the neural cell marker, βIII-tubulin. Isotype-matched antibodies were used as negative controls. ii) NIM-2 consisted of α-MEM with 10 ng/ml bFGF, 10 μM forskolin (Sigma), 25 mM KCl, 2 mM valproic acid and 5 μg/ml insulin. The cells received pre-neural induction in α-MEM medium containing 10% FBS and 10 ng/ml bFGF (Roche) for 24 h [26]. Subsequently, the medium was removed, cells washed with PBS and the NIM#2 added for up to 35 days incubation period with the medium refreshed every 3 days. Cells were monitored continually after neural induction for morphological changes and were lysed for RNA extraction or fixed for immunostaining. The control group received regular medium and harvested at the same time points as the neurogenic group.

### qPCR

The cells were harvested and total RNA isolated using RNeasy mini kit (Qiagen). First strand cDNA from purified RNA was generated using the SuperScript^tm^ III First-Strand synthesis system for qPCR (Invitrogen). The sequence of primers is listed in Supplemental Table I. The LightCycler^®^ 480 SYBR Green I Master (Roch Diagnostic Corp., Indianapolis, IN) was used as described by the manufacturer. Briefly, cDNA template was mixed to PCR Master Mix, with forward/reverse primers and sterile distilled water to a final volume. The reaction mixture was placed in each wells of a 96-well plate, covered and centrifuged at 1000 rpm for 5 minutes at 4°C. The plate was then placed in a LightCycler^®^ 480 II (Roch Diagnostic Corp.) and run for 40 cycles with the following thermal cycling conditions: 95°C for 5 min followed by 40 cycles of 95°C for 10 s, 60°C for 30 s, and 72°C for 10 sec. A relative quantitative analysis method was performed to quantify the relative gene expression compared to the level of the housekeeping gene glyceraldehyde-3-phosphate dehydrogenase (GAPDH).

### Alkaline phosphatase assay

ALP activity of PDLSCs was measured using SensoLyte pNPP ALP Assay Kit (AnaSpec, EGT Corp. Fremont, CA). Briefly, cells were lysed in assay buffer, pelleted and the supernatant collected for measurement of ALP activity. *p*NPP, colorimetric alkaline phosphatase substrate was applied to the sample supernatant and incubated at room temperature for 30 min. Stop Solution was then added and the absorbance was measured at 405 nm. The sample ALP concentration was calculated against standard ALP plots of known concentrations.

### Data analysis

The strategy for the statistical analysis was to test the null hypothesis that the outcome measurements are not different among the groups of interest in each of the experiments. One way ANOVA was used to compare single factor among three or more groups. Two-way ANOVA was used to examine the effects of two independent factors and their interaction effect on the outcome. When data were collected from different donors, mixed-effects model was performed treating donor as a random effect. Shapiro-Wilk test was used to test the normality of the residuals distribution for each model. Log2 transformation was applied when the normality assumption was not met. If the main and/or interaction effect found to be statistically significant, post-hoc comparisons were examined by Tukey HSD (Honestly Significant Difference) test. Adjustment for multiple comparisons were done using Bonferroni method or Benjamini and Hochberg procedure. Values are considered statistically significant when p < 0.05. Data are reported as mean ± SEM. All analyses were performed using JMP^®^ Pro 10.0.0 and SAS 9.3 (SAS Institute Inc., Cary, NC).

## Results

### PDLSC properties on colony formation, population doubling and differentiation potential

**Figure 1:**
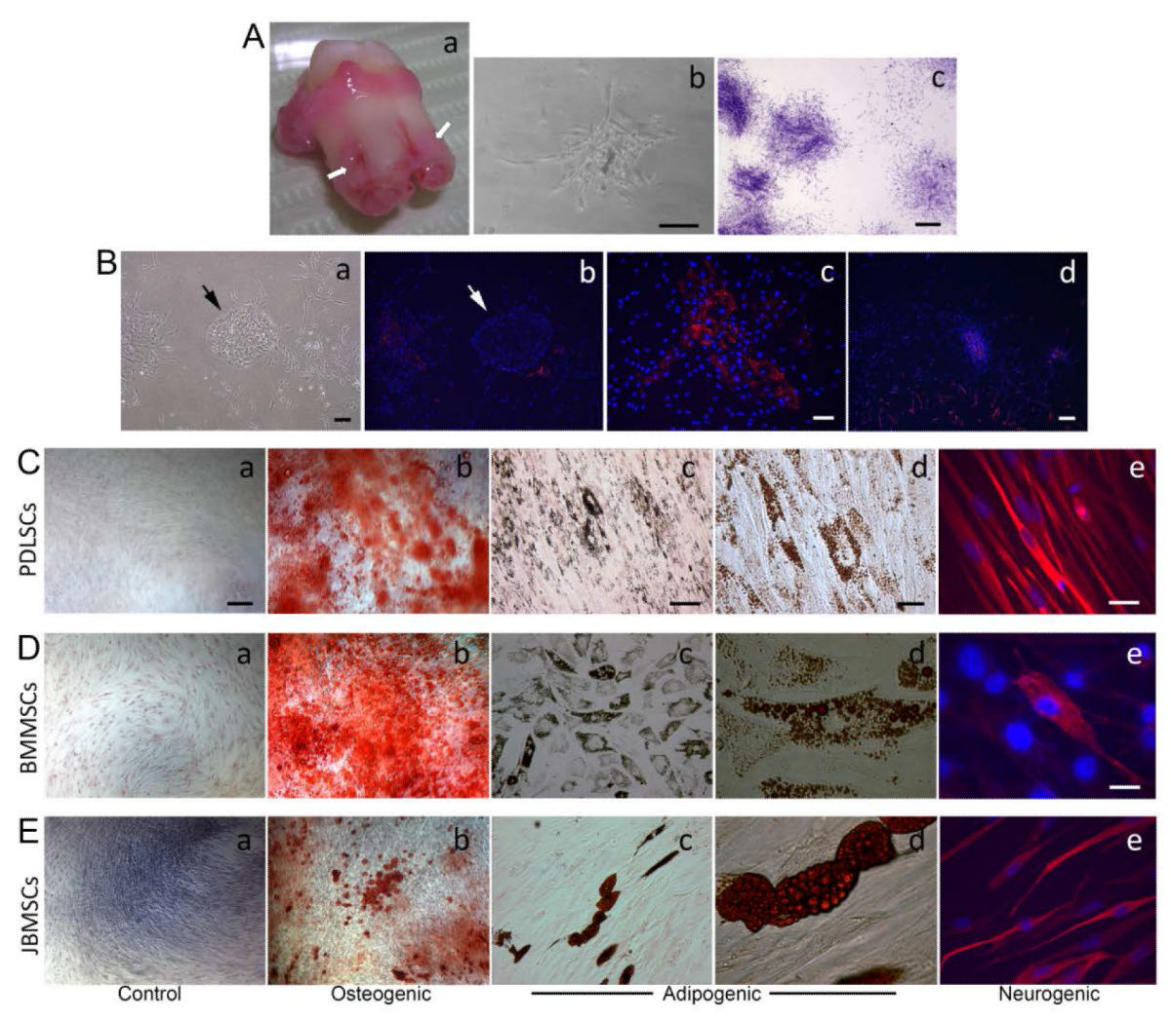
Isolation and characterization of heterogeneous human PDLSCs. (Aa) Image of a freshly extracted third molar. Arrows indicate PDL to be isolated. (Ab) Colony formation after initial seeding (passage 0). (Ac) PDLSC colonies formed at passage 1 fixed and stained with toluidine blue. (Ba) Initial seeding (Passage 0) showing an island of epithelial cells (arrow), a CFU-F (on the left) and scattered single cells. (Bb) STRO-1 staining (red fluorescence) of the culture shown in Ba. Notice the epithelia island is STRO-1 negative. The cell nuclei were stained with DAPI (blue). (Bc) STRO-1 positive in a single PDLSC CFU-F (passage 0). (Bd) STRO-1 positive PDLSC CFU-F and in scattered cells (passage 2). (C-E) Induced differentiation of PDLSCs (C), BMMSCs (D), and JBMSC (E) at passage 2-3. (Ca, Da, Ea) Controlled groups without stimulation; (Cb, Db, Eb) osteogenic induction after ~6 weeks followed by Alizarin Red stain showing mineral deposits; (c & d of C, D, E) adipogenic induction for ~6 weeks followed by Oil-Red stain showing oil droplet-filled adipocyte-like cells; (Ce, De, Ee) neurogenic induction (in NIM-1) for 4 weeks followed by staining of βIII tubulin (red fluorescence). Scale bars: (Ab) 100 μm; (Ac) 1 mm; (Ba, b) 200 μm; (Bc) 100 μm; (a, b of C, D, E) 200 μm; (c of C, D, E) 100 μm; (d of C, d, E) 20 μm; (Ce, Ee) 30 μm; (De) 20 μm.

We first characterized heterogeneous populations (original pool) of PDLSCs isolated from human PDL and seeded in culture plates to observe CFU-Fs as shown in Figure 1Aa,b. These cells were also able to form CFU-F after passaging (Figure 1Ac). To determine the capacity of PDLSCs to expand in vitro, population doubling (PD) studies were carried out. We divided each PDL tissue from the patients into two halves. One half underwent enzyme digestion as described in the Supplemental Materials and Methods, the other half minced into small fragments to allow the PDLSCs to out-grow into the culture plates as described in a previous report [27]. Total PD of cultured heterogeneous population of PDLSCs obtained by digestion reached 51.2 ± 13.0 (mean ± SEM) ranging from 28.3 – 93.4 based on four samples from four patients (aged 20-26). PDLSCs obtained by out-growth reached the PD of 54.8 ± 9.3 ranging from 24.4 – 72.8. There was no statistically significant difference in the PDs between PDLSCs obtained from different methods (Supplementary Figure 1), suggesting that the two isolation methods did not cause a difference in the cell life span in cultures. In order to observe CFU formation, we then determined to use enzyme digestion method to isolate PDLSCs used for all the experiments in the present study.

At passage 0, epithelial cell islands were frequently observed (Figure 1Ba,b). These epithelial cells are expanded from the epithelial cell rests of Malassez residing in the PDL [28]. STRO-1^+^ cells were normally detected in the CFU-F and tended to be located in the center of the colony (Figure 1Bc). They were also detected scattering around the epithelial islands and were not necessarily associated with CFU-Fs (Figure 1Bb, d).

PDLSCs at passages 2-3 were subjected to osteogenic, adipogenic or neurogenic induction. BMMSCs and JBMSCs were used as a comparison. As demonstrated in Figure 1C- E, heterogeneous PDLSCs are multipotent capable of giving rise to the three lineages following respective stimulation. Under ~6 weeks of osteogenic stimulus, cell cultures formed mineral deposits indicated by Alizarin Red stain (Figure 1Cb, Db, Eb); after ~6 weeks of adipogenic stimulus, cells accumulated oil droplets revealed by Oil-red stain (Figure 1 Cc,d; Dc,d; Ec,d); and after 4 weeks of neurogenic stimulus, cells were stained positively with the neural marker βIII tubulin (Figure 1 Ce, De, Ee).

### Heterogeneous PDLSCs expressed mesenchymal stem cell markers

**Figure 2:**
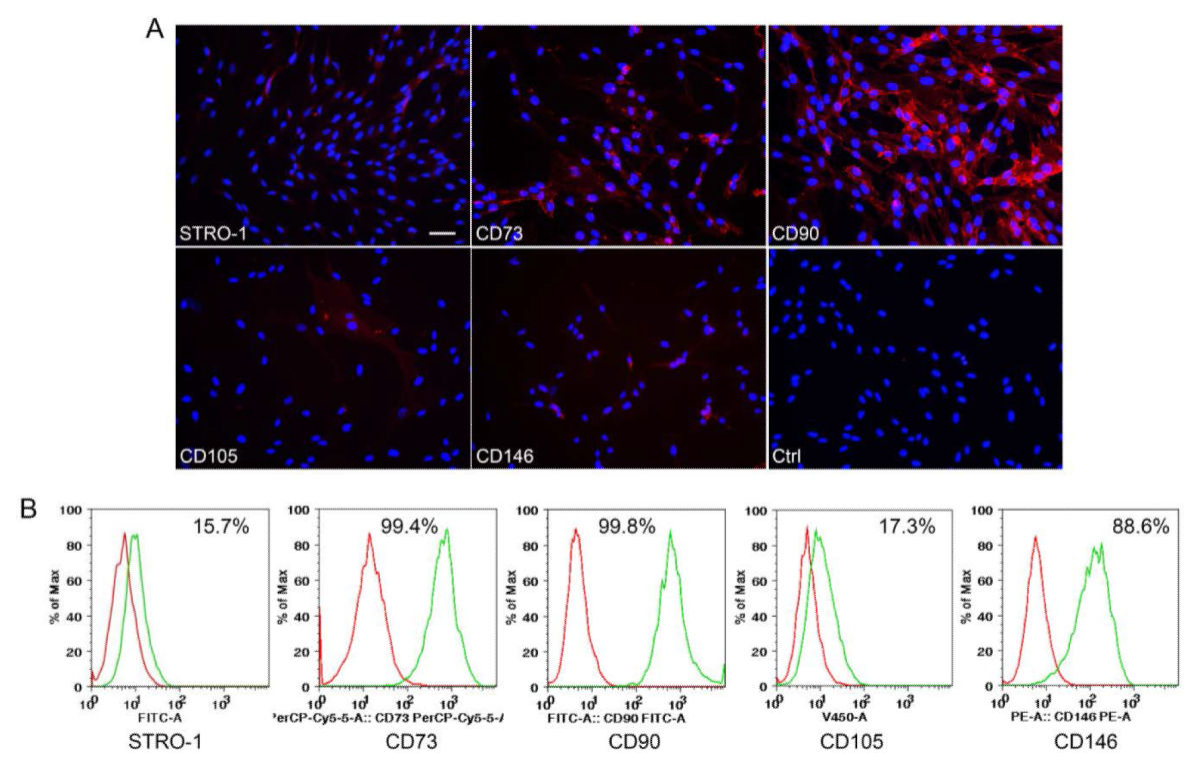
Marker expression by heterogeneous PDLSCs. (A) Immunocytofluorescence staining of STRO-1, CD73, CD90, CD106 and CD146. Subconfluent cells were all at passage 1. Scale bars: (A) 50 μm for all marker genes and isotype control (Ctrl). (B) Flow cytometry analysis of cell surface markers and the percentage of positive cells with marker expression in the heterogeneous PDLSC population at passage 3 (red peak: control; green peak: specific antibody detection).

We further detected the expression of MSC markers STRO-1, CD73, CD90, CD105 and CD146 for PDLSCs at passage 1 using immunocytofluorescence. As shown in Figure 2A, STRO-1 and all CD markers tested were positive. In CFU-F cultures, we also counted the percentage of each MSC marker expressing cells within each colony to be as follows -- STRO-1: 20-30%; CD73: ~70%; CD90: ~100%; CD105: ~5%; CD146: ~51%. Flow cytometry analysis after cell expansion (at passage 3) indicated the percentage of cells in the entire population expressing these markers, as depicted in Figure 2B showing high percentages of CD73, CD90 and CD146 and relatively low percentages of STRO-1 and CD105.

### Profiles of mesenchymal stem cell marker of ALP^+^ PDLSC subpopulation

**Figure 3:**
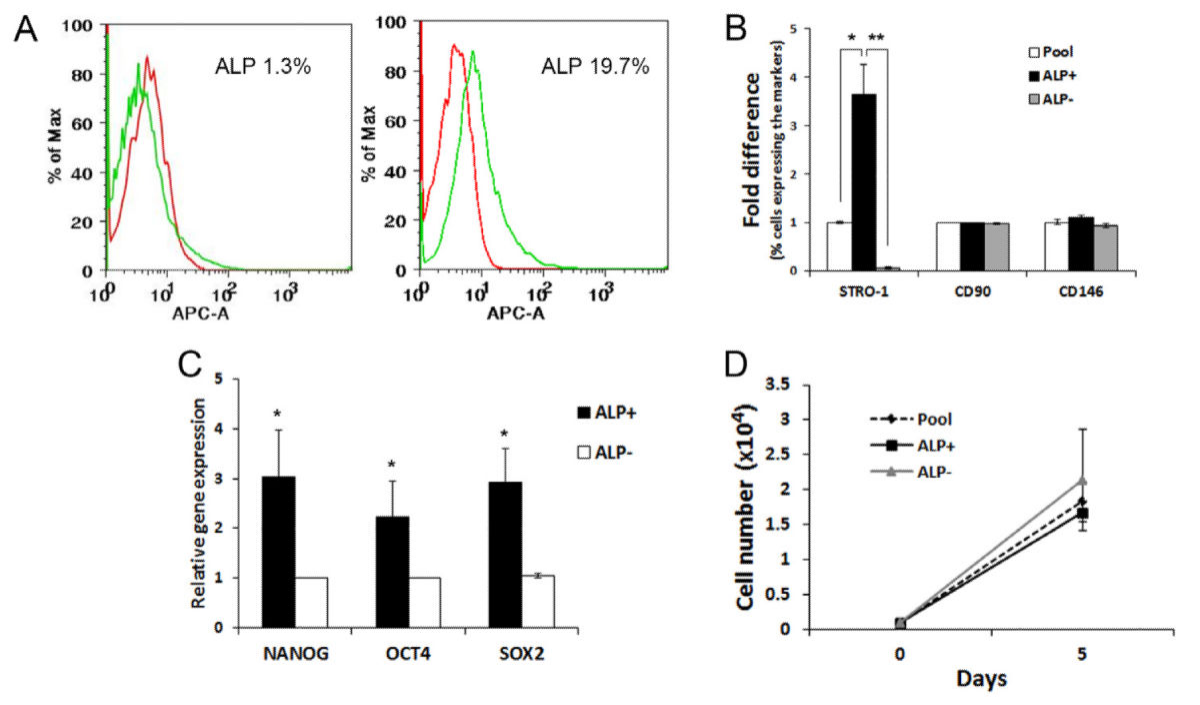
Characterization of the early passage ALP subpopulation of PDLSCs. (A) Flow cytometry analysis of ALP subpopulation in PDLSCs at passages 2-3. Representative data showing a wide variation of percentages of the ALP^+^ subpopulation, with as little as 1.3% or up to 19.7%. (B) Flow cytometry analysis of the quadruple staining of STRO-1, CD90, CD146 and ALP showing the levels of STRO-1^+^, CD90^+^ and CD146^+^ cells within the subpopulations of sorted ALP^+^ and ALP^−^ compared to the percentage of those markers in the original entire population (pool). (One-way ANOVA was performed to compare the means percentage among three populations. Tukey HSD was used to determine the pair difference. Significantly different, p<0.05*; p<0.0001**; n=6). Original expression levels in percentage presented in supplemental Table II. Cells analyzed at passages 1-3. (C) Stemness gene expression level between ALP^+^ and ALP^−^ subpopulations. (Based on 4 independent experiments each from a different donor and tested in triplicate). (Significantly different between each ALP^+^/ALP^−^ pair, p<0.05*. The subpopulation effect for each gene was tested using a mixed-effects model.) (D) Growth rate of the pool, ALP^+^ and ALP^−^ PDLSCs. Cells were seeded at 0.1 × 10^4^ per well of 12-well plates in triplicate on day 0 and harvested/counted on day 5. Data based on 3 independent experiments (3 different donors), each assayed in duplicate. No significant difference was found among the subpopulation groups using a mixed-effects model (*p* = 0.42).

We performed quadruple staining against ALP, STRO-1, CD90 and CD146 and analyzed by flow cytometry. With such an approach, we were able to obtain the following results. Cells at low passages of 1-3, ALP^+^ cells constituted 0.1-19.7% (Mean ± SEM, 7.3 ± 1.8) of the total PDLSC pools based on 14 samples each from a different donor and the representative data are presented in Figure 3A. We then asked whether ALP^+^ PDLSC subpopulation presents different levels of MSC makers compared to those in the original pool of PDLSCs or ALP^−^ cells. As shown in Figure 3B and Supplemental Table II, the percentage of cells expressing STRO-1^+^ in the ALP^+^ subpopulation was 3 times more compared to the original PDLSC pool and 71 times more compared to the ALP^−^ subpopulation.

### Stemness gene profiles, proliferation and differentiation capacity of PDLSC ALP^+^/ALP^−^ subpopulations at low passages

Since ALP^+^ PDLSCs expressed higher percentage of STRO-1, we asked whether ALP^+^ and ALP^−^ cells express different levels of stemness genes *OCT4, NANOG* and *SOX2* and whether they grow at a different rate in cultures. We first used cells of low passages (p2-3) to examine these conditions. Samples from 4 donors examined, ALP^+^ cells expressed higher levels of stemness genes (Figure 3C) (p<0.05). Out of samples tested from 3 donors, two showed a slightly slower growth in ALP^+^ than ALP^−^ cells, while that of the third donor ALP^+^ had a faster growth; thus no statistically significant difference was shown (Figure 3D).

We next tested whether there was a difference in multi-potent differentiation capacities between ALP^+^ and ALP^−^ subpopulations. We found that ALP^+^ and ALP^−^ cells showed similar osteogenic, chondrogenic and neurogenic potential (Supplemental Figures 2 and 3) with minor differences in gene induction at different time points after stimulation. While no observable difference in the amount of mineral deposits in culture after osteogenesis can be noted between ALP^+^ and ALP^−^ groups, difference in gene induction was noted. ALP and BSP expression at week 2 was not induced in ALP^−^ group, and OCN expression was not increased at week 1 in ALP^−^ group, compared to the ALP^+^ group (Supplemental Figure 2B). Gene expression was not examined for chondrogenesis due to low number of ALP^+^ cells and other cell groups had to match this cell number for comparison. For this reason, pellet culture experiments were affected. The Alcian blue staining of the monolayer cultures did not detect observable difference between ALP^+^ and ALP^−^ cells (Supplemental Figure 3Aa). Few successful pellet cultures showed lack of difference between ALP^+^ and Pool cells (Supplemental Figure 3Ab). In neurogenesis, no difference can be noted from the βIII tubulin staining between groups (Supplemental Figure 3Ba). qPCR data showed that CNPase, Nav1.6 and NF1 expression was decreased or not induced in ALP^−^ group compared to ALP^+^ group (Supplemental Figure 3Bb).

**Figure 4:**
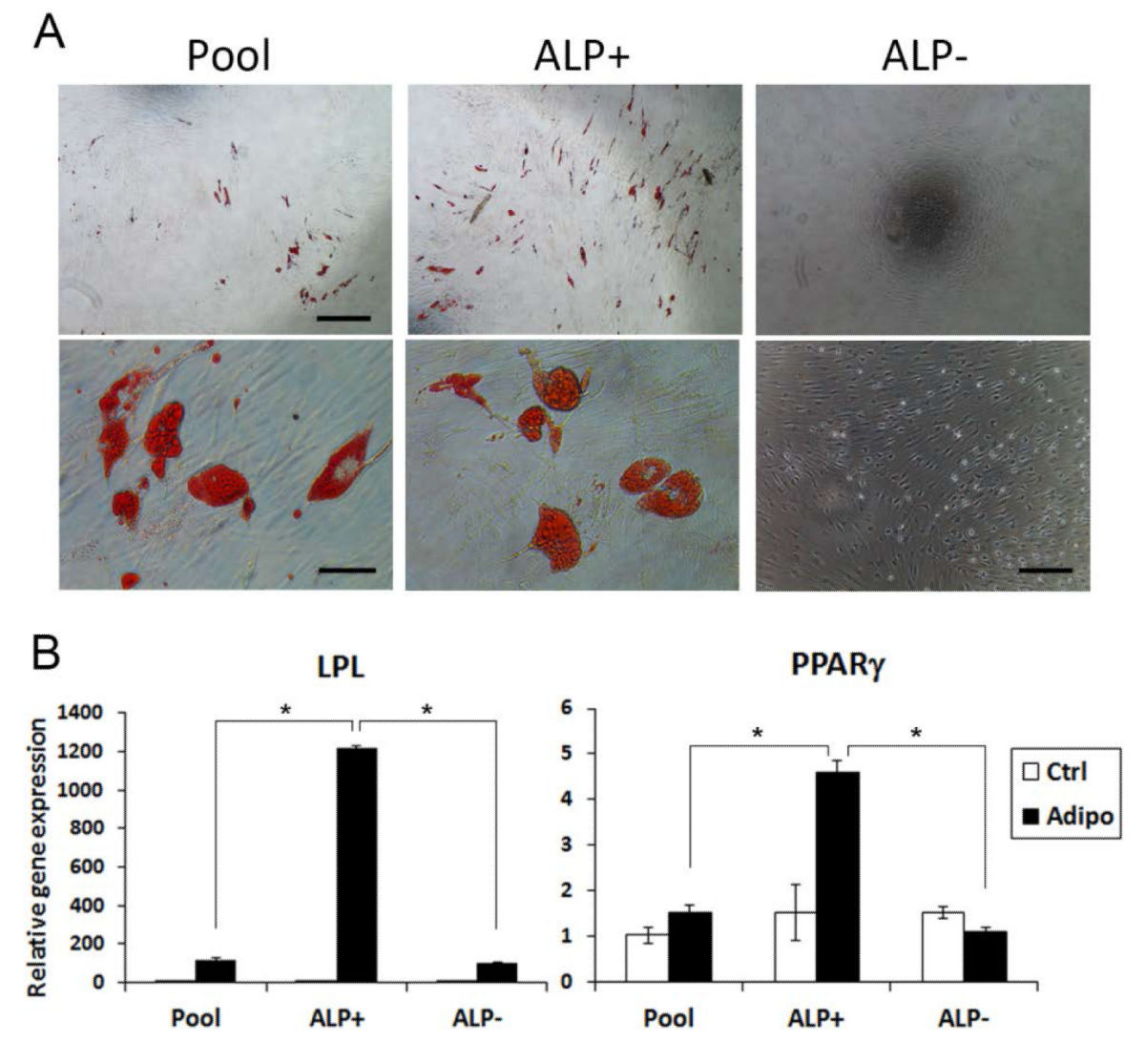
Adipogenic differentiation capacities of PDLSC subpopulations. Sorted PDLSCs at passages 2-3 were immediately seeded at a density of 2×10^4^ cells/well of 24-well plates or 1×10^4^ cells/well of 48-well plates and after reaching ~70% confluence they were stimulated under the adipogenic condition. (A) Adipogenic induction of cells for 8 weeks and stained with Oil Red. Scale bars: top three images, 500 μm; bottom left and middle images, 50 μm; bottom right image, 200 μm. (B) qPCR of *LPL* and *PPARγ* in cells under adipogenic induction for 3 weeks, representative data from one donor measured in triplicate. (Significantly different, p≤0.0001*. Two-way ANOVA was performed for each gene to test the subpopulation, adipogenic condition, and their interaction effects on the gene expression. Post-hoc analysis using Tukey HSD was performed to identify the specific pairs of difference).

A drastic difference between ALP^+^ and ALP^−^ cells was found in adipogenesis that ALP^−^ cells were incapable of undergoing adipogenic differentiation (Figure 4). ALP^+^ cells had higher number of cells that stained positively with Oil-red than the pool and ALP^−^ groups (Figure 4A). Additionally, adipogenic markers LPL and PPARγ were much more induced in ALP^+^ cells compared to either the original pool of PDLSCs or the ALP^−^ cells (Figure 4B).

### Expression of stemness genes and ALP at higher cell passages

**Figure 5:**
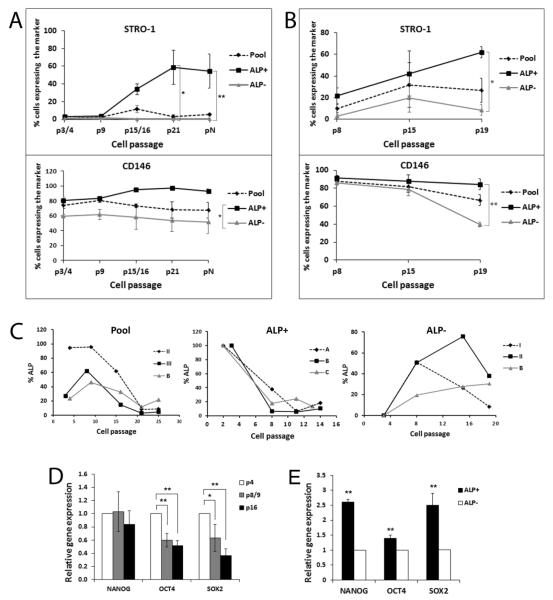
Expression of stemness genes by subpopulations of PDLSCs at higher passages. (A) PDLSC original pool was continuously cultured and passaged (1:3 ratio/dilution) at subconfluence and subjected to flow cytometry analysis following quadruple staining of STRO-1, CD90. CD146 and ALP. The expression of STRO-1 and CD146 of the pool and subpopulations at different passages was plotted. pN, at the cell passage axis indicates that cells were passaged at passage 8 with 1:15 ratio/dilution and continued to passage in this manner until reaching passage 16 (counting from passage 8). p3/4 or p15/16 denotes that cells were analyzed either at passage 3 or 4, or passage 15 or 16, respectively. Data represent mean ± SEM of cells from three different donors. (B) PDLSC ALP^−^ subpopulation was sorted from original pool PDLSCs at passage 3 and were continuously cultured and passaged (1:3 ratio/dilution) at subconfluence and subjected to flow cytometry analysis with the same approach as for (A). Data represent mean ± SEM of cells from three different donors. (C) PDLSC original pools, ALP^+^ subpopulation, and ALP^−^ subpopulation were continuously passaged (1:3 ratio/dilution) and subjected to flow cytometry analysis to detect the percentages of the cells expressing ALP. Each graph presents data of cells from three different donors (A, B, C, I, II, II are donor codes). ALP^+^ subpopulation was isolated at passages 2-3 and the ALP levels were considered as 100% right after sorting. ALP^−^ subpopulation was isolated at passage 3 and considered as 0% ALP right after sorting. (D) Expression of stemness associated genes *NANOG, OCT4* and *SOX2* by PDLSC pools at various passages detected by qPCR (Data represent mean ± SEM from three different donors each assayed in triplicate). (E) Expression of stemness associated genes *NANOG, OCT4* and *SOX2* by PDLSC subpopulations ALP^+^ and ALP^−^ at passage 8. PDLSC pools were first sorted at passages 2-3 and the ALP^−^ subpopulation was continuously cultured and passaged until passage 8 and sorted again to separate ALP^+^ and ALP^−^ for qPCR analysis. (Data represent mean ± SEM from two different donors each assayed in triplicate.) A mixed-effects model with repeated measures was performed for each gene in (A), (B), and (D) with Tukey HSD post-hoc analysis. A mixed effect model was used to test the subpopulation effect on the expression of each gene in randomly selected patients (E). (Significantly different, p<0.05*; p<0.0001**).

We then asked whether the expression of ALP, STRO-1, CD146, *NANOG, OCT4* and *SOX2* changes at higher cell passages. From the flow cytometry analysis of STRO-1 and CD146, both markers were higher in the ALP^+^ subpopulation than ALP^−^ throughout passaging, particularly at later passages (Figure 5A, B). Data shown in Figure 5A are from experiments that original pool of PDLSCs were passaged and underwent quadruple staining (STRO-1, CD90, CD146 and ALP) and the STRO-1 and CD146 expression analyzed at various cell passages. Data in Figure 5B are from those that ALP^−^ subpopulation were sorted out from the pool and continued to be passaged and analyzed with flow cytometry following quadruple staining as the above. The data showed that STRO-1 and CD146 both are higher in ALP^+^ than in ALP^−^ cells (Figure 5A, B; p<0.05 or p<0.0001). The findings indicate that both STRO-1 and CD146 are expressed at higher levels in ALP^+^ cells either in the Pool population (Figure 5A) or in ALP^+^ that emerged from the ALP^−^ subpopulation (Figure 5B) during passaging. Surprisingly, STRO-1 expression levels appeared to sustain the cell passaging and increased in the ALP^+^ subpopulation even up to >passage 19. CD146 also remained high in older passages especially in the ALP^+^ subpopulation (Figure 5B).

We further examined ALP levels in cells from low to high passages. PDLSCs from three donors were analyzed individually and data are presented in Figure 5C. The Pool group showed that ALP levels fluctuated from lower to higher passages and dwindled down after passage 20. Donor II had exceptionally high ALP levels until passage 20. The sorted ALP^+^ subpopulation at low passages 2-3 were continuously passaged and the level dropped to a steady level and sustained until the last measurement at passages 13/14. The sorted ALP^−^ subpopulation at passage 3 showed re-emergence of the ALP^+^ subpopulation as cells continued to be passaged. The ALP levels increased from 0% to as high as 76% in donor II and the level decreased as passages reaching 19. Cells from donor B, however, continued to show increased ALP^+^ cells.

The stemness associated genes *NANOG, OCT4* and *SOX2* were also examined for their expression levels using qPCR and the data are shown in Figure 5D. Besides *NANOG* which appeared to sustain at the similar levels at up to passage 16, *OCT4* and especially *SOX2* dropped as cells reaching higher passages. To correlate the stemness associated gene levels to ALP^+^ and ALP^−^ subpopulations, Pool PDLSCs were sorted at passages 2/3 and the sorted ALP^−^ subpopulation were continuously passaged until passage 8 and sorted again to separate ALP^+^/ALP^−^ subpopulations and the stemness associated gene expression levels examined by qPCR. The data in Figure 5E show that the re-emerged ALP^+^ population from ALP^−^ cells possessed higher stemness gene expression levels than ALP^−^ (p<0.05 or p<0.0001).

### Effects of over-confluence and vitamin C treatment on ALP and stemness gene expression

**Figure 6:**
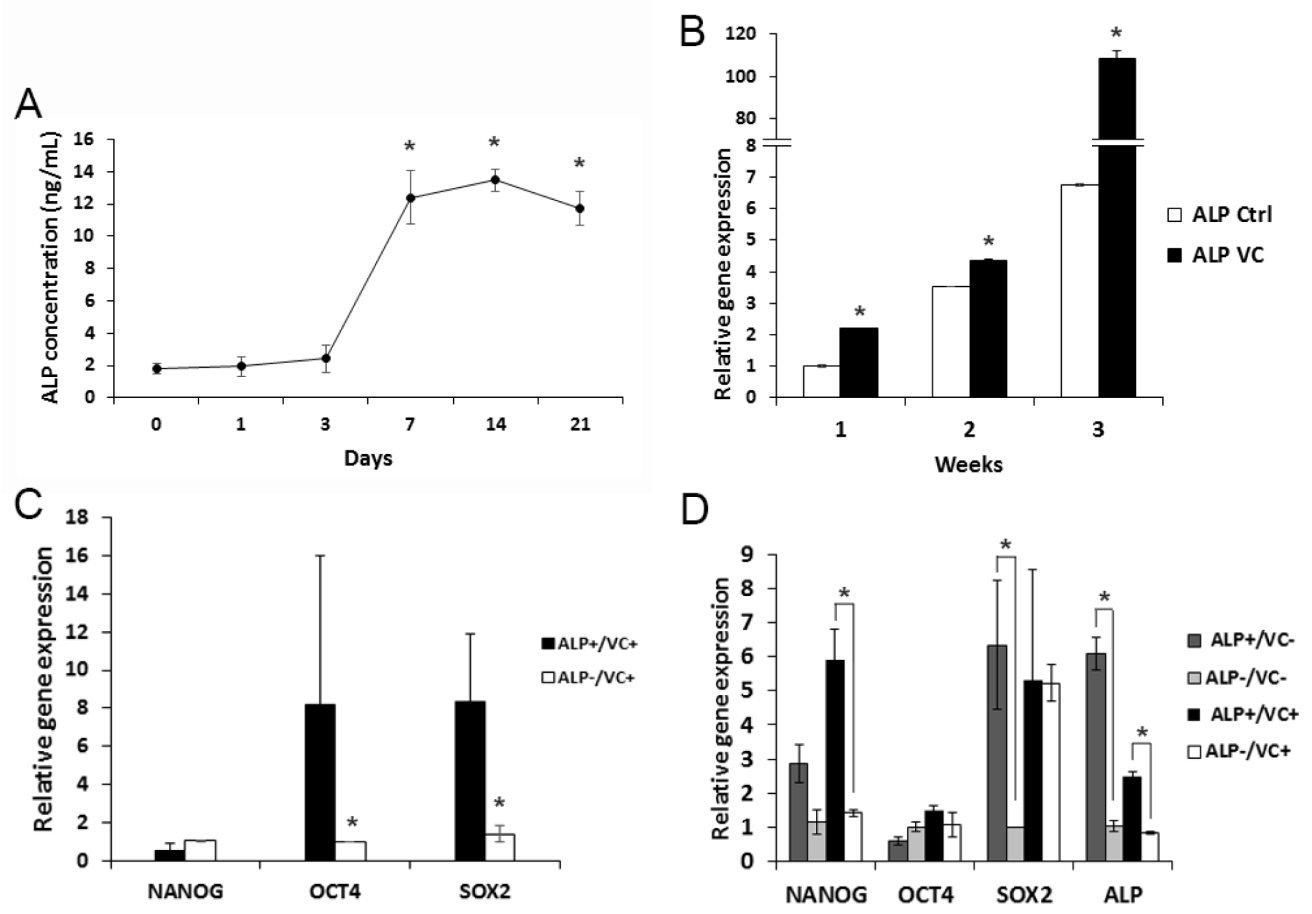
ALP^+^ PDLSCs after confluence and vitamin C treatment. (A) PDLSCs at passages 2-4 were seeded into wells of 6-well plates and allowed to grow confluent for up to 21 days. At different time points, cells were subjected to ALP assay. Data represent mean ± SEM from three different donors each assayed in triplicate. A mixed effect model was used to test the effect of time on protein levels. All pairwise comparisons were done and p-values were adjusted using the Tukey method. (Significantly different, p≤0.0001*). (B) PDLSCs at passage 3 were seeded in wells of 6 well-plates and vitamin C (VC) at 20 μg/mL was added to experimental group at ~80% subconfluence. At different time points, cells were harvested and subjected to qPCR detection of ALP expression. Data represent one donor assayed in triplicate. Two sample t-tests were used and p-values were adjusted using Bonferroni method. (Significantly different, p<0.0001*). (C) PDLSCs were cultured with VC for 1 week and sorted into ALP^+^ and ALP^−^ cells for RNA isolation and qPCR analysis of the stemness gene expression. Data represent mean ± SEM from three different donors each assayed in triplicate. (The qPCR data values were very low in these cells and some of them were below detectable levels). A mixed effect model was used to test the subpopulation effect on the expression of each gene in randomly selected donors. The p-values were adjusted using the Benjamini-Hochberg procedure. (Significantly different, p=0.045*). (D) PDLSCs were cultured with or without VC for 3 weeks and cells were sorted into ALP^+^ and ALP^−^ cells for RNA isolation and qPCR analysis. Data represent one donor assayed in triplicate. (Significantly different, p≤0.05*). (70-99% of cells were ALP^+^ after sorting in C, D). PDLSCs were grown in medium absent of L-ascorbic acid 2-phospate for the VC experiments. A linear model with subpopulation as the main effect (ALP^+^ and ALP^−^) was used for each gene with or without VC. P-values were adjusted using the false discovery rate procedure.

All the above ALP assays were performed when PDLSCs were grown at subconfluence. We then examined ALP expression after cells were over-confluent, we found that ALP expression increased over time reaching the peak after 7 days (Figure 6A). ALP levels were significantly higher after 7 days compared to that at day 0 or 3, while no difference was found from 7 days to 21 days. ALP levels further increased if cells were treated with vitamin C (VC) as shown in Figure 6B, especially after 3 weeks compared to the control group. We then asked whether the stemness gene expression between ALP^+^ and ALP^−^ cells was changed. Data in Figure 6C and D indicate that after VC treatment, the stemness gene expression levels in ALP^+^ cells were minimally or no longer higher than those in ALP^−^ cells especially after 3 weeks of treatment (Figure 6D). Nonetheless, ALP^+^ cells appeared to maintain the propensity to express slightly higher stemness genes, although difficult to detect the difference in some groups. ALP gene expression was higher in ALP^+^ cells than in ALP^−^ cells indicating that the selection of ALP^+^ cells correlated to their gene levels.

## Discussion

Our present study has identified several key features of hPDLSCs in vitro: i) ALP expression is associated with higher levels of stemness gene expression. ii) Both ALP^+^ and ALP^−^ cells are multipotent except that ALP^−^ cells lack adipogenic potential. iii) ALP^+^ cells maintain higher levels of STRO-1, CD146 and *NANOG* expression than ALP^−^ cells in cultures even after a high number of passaging.

The maintenance of stemness of cultured adult stem cells has been studied from various perspectives. One difficulty for such studies is the variation among subtypes of MSCs and the lack of a set of universal stemness markers to identify them. Subtypes of MSCs in different mesenchymal tissues are subtly different in their lineage commitment, multi-lineage differentiation potential and in vitro proliferative capacity [6]. Another issue is cell passaging of the in vitro study. Many assays are difficult to perform to study subpopulations at passage 0 due to low cell numbers. We used low passages 2-3 to perform the subpopulation separation. For cell sorting, we needed 0.5-1 × 10^7^ of cells rendering the use of passage 0 technically prohibitive. Another difficulty is the frequent low percentage of ALP^+^ cells among the cell populations at low passages or subconfluence.

Treating MSCs such as stem cells from apical papilla (SCAP), another type of dental stem cells, with growth factors such as bFGF increases the expression of STRO-1, *NANOG, OCT4, SOX2* and *REX1* and increases the proliferation while down-regulating the differentiation [19]. Such enhanced expression of stemness genes and inhibition of cell differentiation in vitro is considered a boost of stem cell stemness. On the other hand, treating DPSCs with small molecules such as SC1 and rapamycin decreased cell proliferation although the expression of the stemness genes *NANOG, OCT4* and *SOX2* is up-regulated and the differentiation down-regulated [18]. There appears to have contradictory findings in the relationship between stemness and proliferation rate in vitro. The issue lies in the definition of self-renewal and proliferation when cells are grown in cultures. Stem cell renewal and progenitor cell clonal amplification is a different stage of cell status, although both go through cell cycle/division. Sacchetti et al. identified a subpopulation CD146^+^ BMMSCs that are modulated in opposite ways [21]. bFGF increases cell proliferation and attenuates the stem cell phenotype, while TGF-β inhibits proliferation and preserves or enhances the stemness. Their findings suggest that enhanced proliferation pushes BMMSCs toward more differentiated progenitor cells rather than maintained as stem cells which manifest as slow proliferating in cultures. The association of ALP^+^ PDLSCs with higher expression of STRO-1, CD146, *NANOG, OCT4* and *SOX2* suggests that these are a more immature phenotype than the ALP^−^ PDLSCs. ALP^+^ cells do not have a higher proliferation rate, some cases lower, than ALP^−^ cells which may indicate a slight link to the situation of the small molecule treated DPSCs or TGF-β treated BMMSCs. Because ALP is a marker for the undifferentiated state of pluripotent stem cells [16,17], our findings on the association of PDLSC ALP^+^ subpopulation with the higher expression of stemness genes indicate that such phenomenon also applies to adult stem cells in the case of PDLSCs. Additionally, ALP^+^ cells maintained higher expression of STRO-1, *NANOG*, *OCT4* and *SOX2* than ALP^−^ cells at high passages, which also supports the stemness status of ALP^+^ subpopulation. This is in contrast to BMMSCs that ALP^−^ cells have more multipotential capacity and express higher pluripotent marker genes REX1 and NANOG than ALP^+^ [15].

Regarding that lack of adipogenic potential of ALP^−^ PDLSCs, two mechanisms may be involved. One is that ALP^−^ subpopulation is less immature than ALP^+^. BMMSCs lose chondrogenesis and adipogenesis after passaging whereas osteogenesis is the last differentiation potency to lose [29]. The other is that ALP is involved in the control of intracellular lipid accumulation in preadipocyte maturation [30,31], therefore, absence of ALP may prevent formation of lipid in cells.

Our studies also showed that ALP^+^ is transient in the pool of PDLSCs evidenced by the finding that the percentage of ALP^+^ cells in the sorted ALP^+^ subpopulation came down after cell passaging while the ALP^+^ subpopulation also re-emerged from sorted ALP^−^ cells. This ALP^+^ subpopulation either in the original PDLSC pool or emerged from ALP^−^ subpopulation at low or high passages expressed higher levels of stemness genes than the ALP^−^ subpopulation. At low passages 1-3, ALP^+^ cells constituted 0.1-19.7% of the total PDLSC pools based on 14 samples each from a different donor. There was a cell sample from another donor expressing ALP as high as 94.5% measured at passage 4 (Figure 5C, Pool Group, donor II) and the ALP levels stayed high until passage 21. The sorted ALP^−^ subpopulation from this sample also showed a drastic re-emergence of ALP^+^ subpopulation upon further cell culturing and passaging (Figure 5C, ALP^−^ Group, donor II). This sample was exceptional among all samples we tested, indicating that potentially, although less common, PDLSCs from some donors may contain very high percentage of ALP^+^ cells.

The re-emergence of ALP^+^ from ALP^−^ cells and their expression of higher level of stemness genes than ALP^−^ cells indicates that PLDSCs appear to contain subpopulations that remain immature in cultures after long-term passaging and ALP is a marker for such type of subpopulations. Recent studies have shown that human PDLSCs contain subpopulation connexin 43^+^ cells that are highly potent capable of forming teratomas in vivo with tissues representing all three germ layers [32]. This could also be due to an in vitro culturing phenomenon. Pierantozzi et al. [33] have shown that *NANOG* expression in MSCs increased after culturing. Several findings in our present studies may explain these phenomena: i) ALP^+^ cells are always associated with higher levels of *NANOG, OCT4* and *SOX2* than ALP^−^ cells (Figure 3C, 5E); ii) *NANOG* appeared to sustain high levels after passaging (Figure 5D), and iii) PDLSCs maintain high levels of STRO-1, CD146 at high passages. It is likely that PDLSCs maintain their stem/progenitor status by having subpopulation of cells capable of reversing to or remaining at the immature stage. Another possibility is the cell cycle-dependent ALP expression identified in the studies of human bone cells [34]. However, the relationship of this cell cycle dependence to ALP association with stemness is not clear.

When PDLSCs cells were grown in conditions that favor cell differentiation such as over-confluence or VC treatment, ALP^+^ subpopulation and its levels increased, which is similar to bone cells [34]. Interestingly, despite what was anticipated that under differentiation conducive conditions, stemness genes expressed by ALP^+^ PDLSCs seemed to retain some potential to be higher than the ALP^−^ subpopulation, albeit minimal; unlike bone derived cells that ALP^+^ subpopulation is more associated with more differentiated state than ALP^−^ counterparts [14]. In conclusion, our present studies revealed the dynamic ALP expression and its relationship to the stemness properties of the PDLSC subpopulations. Further studies are needed to understand the roles of ALP association with stemness in the PDLSC subpopulation and this relationship to ALP association with other commitment such as osteogenesis or cementogenesis.

## Supplementary Material

Supplementary material

Supplementary_figure

Supplementary_table_1

Supplementary_table_1

## Abbreviations

hPDLSCs: Human Periodontal Ligament Stem Cells
PDL: Periodontal Ligament
DPSCs: Dental Pulp Stem Cells
MSC: Mesenchymal Stromal/stem Cells
ALP: Alkaline Phosphatase
CFU-F: Colony Formation Units of Fibroblastic Cells
BSP: Bone Sialophospho-protein
OCN: Osteocalcin
RUNX2: Runt-related Transcription Factor 2
LPL: Lipoprotein Lipase
PPARγ2: Peroxisome Proliferator-activated Receptor Gamma 2
NFM: Neurofilament M
CNPase: 2′, 3′-cyclic nucleotide-3′-phosphodiesterase
GFAP: Glial Fibrillary Acidic Protein
NF1: Neurofibromin 1
Nav1.6: Sodium Channel SCN8A
