# Supplementary material for: Differential Properties of Human ALP+ Periodontal Ligament Stem Cells vs Their ALP- Counterparts
Source: J Stem Cell Res Ther. Author manuscript; Available in PMC 2016 Jan 20. (PMC4720165; doi:10.4172/2157-7633.1000292)
Supplement: Supplementary material [file NIHMS738577-supplement-Supplementary_material.docx]

**Supplemental Materials and Methods**

Population Doubling (PD)

To study growth difference of PDL cells isolated either by enzyme digestion or explant outgrowth, some PDL samples was divided into two halves to isolate PDLSCs by enzyme digestion (PDLSCs-d) or explant outgrowth method (PDLSCs-o). Pool of PDLSCs either isolated using enzyme digestion or explant outgrowth were seeded at low density (~60 cells/CM^2^) and allowed to grow until ~70-80% confluence. Cells were then passaged and seeded at the same cell density, i.e., ~60 cells/CM^2^. This cell passaging was at splitting ratios of approximately 1:200 to 1:800. The population doubling was calculated at every passage based on our previous report [[1](#_ENREF_1)]. To determine finite population doublings, cumulative addition of total numbers were generated from each passage until the cells ceased dividing. The criterion for cell senescence was that the cells did not divide for a month in culture.

Reference

1. Huang GTJ, Shagramanova K, Chan SW (2006) **Formation of odontoblast-like cells from cultured human dental pulp cells on dentin in vitro.** J Endod **32:**1066-1073.
